# Identification of Volatile Markers of Colorectal Cancer from Tumor Tissues Using Volatilomic Approach

**DOI:** 10.3390/molecules28165990

**Published:** 2023-08-10

**Authors:** Linda Mezmale, Marcis Leja, Anna Marija Lescinska, Andrejs Pčolkins, Elina Kononova, Inga Bogdanova, Inese Polaka, Ilmars Stonans, Arnis Kirsners, Clemens Ager, Pawel Mochalski

**Affiliations:** 1 Institute of Clinical and Preventive Medicine, Faculty of Medicine, University of Latvia, LV-1586 Riga, Latvia; marcis.leja@lu.lv (M.L.); anna_marija.lescinska@lu.lv (A.M.L.); andrejs.pcholkins@gmail.com (A.P.); elina.kononova@lu.lv (E.K.); inga.bogdanova@lu.lv (I.B.); inese.polaka@lu.lv (I.P.); ilmars.stonans@lu.lv (I.S.); pawel.mochalski@ujk.edu.pl (P.M.); 2 Riga East University Hospital, LV-1038 Riga, Latvia; 3 Faculty of Residency, Riga Stradins University, LV-1007 Riga, Latvia; 4 Digestive Diseases Centre GASTRO, LV-1079 Riga, Latvia; 5 Institute for Breath Research, University of Innsbruck, 6020 Dornbirn, Austria; clemens.ager@a1.net; 6 Institute of Chemistry, Jan Kochanowski University of Kielce, 25-369 Kielce, Poland

**Keywords:** volatile organic compounds, colorectal cancer, gas chromatography-mass spectrometry, colorectal tissue

## Abstract

The human body releases numerous volatile organic compounds (VOCs) through tissues and various body fluids, including breath. These compounds form a specific chemical profile that may be used to detect the colorectal cancer CRC-related changes in human metabolism and thereby diagnose this type of cancer. The main goal of this study was to investigate the volatile signatures formed by VOCs released from the CRC tissue. For this purpose, headspace solid-phase microextraction gas chromatography-mass spectrometry was applied. In total, 163 compounds were detected. Both cancerous and non-cancerous tissues emitted 138 common VOCs. Ten volatiles (2-butanone; dodecane; benzaldehyde; pyridine; octane; 2-pentanone; toluene; p-xylene; n-pentane; 2-methyl-2-propanol) occurred in at least 90% of both types of samples; 1-propanol in cancer tissue (86% in normal one), acetone in normal tissue (82% in cancer one). Four compounds (1-propanol, pyridine, isoprene, methyl thiolacetate) were found to have increased emissions from cancer tissue, whereas eleven showed reduced release from this type of tissue (2-butanone; 2-pentanone; 2-methyl-2-propanol; ethyl acetate; 3-methyl-1-butanol; d-limonene; tetradecane; dodecanal; tridecane; 2-ethyl-1-hexanol; cyclohexanone). The outcomes of this study provide evidence that the VOCs signature of the CRC tissue is altered by the CRC. The volatile constituents of this distinct signature can be emitted through exhalation and serve as potential biomarkers for identifying the presence of CRC. Reliable identification of the VOCs associated with CRC is essential to guide and tune the development of advanced sensor technologies that can effectively and sensitively detect and quantify these markers.

## 1. Introduction

Colorectal cancer (CRC) is a significant public health concern. This type of cancer is the third most often diagnosed cancer and the second leading cause of cancer death worldwide. In 2020, there were an estimated 1.9 million new cases of CRC and 935,000 deaths globally [1]. However, the 5-years survival rate could be increased up to 90% if CRC is diagnosed early [2]. The improvement of patient outcomes and the reduction in mortality rates heavily rely on early detection. Hence, in any national health system program, it is crucial to prioritize the development of a dependable and efficient screening tool. The screening modalities for CRC do exist and are recommended for routine clinical applications in most of the developed countries. Current screening methods, such as colonoscopy, fecal immunochemical test (FIT), and stool deoxyribonucleic acid (DNA) test are effective but participation in these public screening programs is low. There are several reasons for it: the test may be linked to psychological or physical discomfort, cost, and inconvenience; in addition, the accuracy of the available test could be improved [3]. Therefore, researchers have been exploring alternative screening methods that are less invasive and more convenient.

In recent years, the analysis of volatile organic compounds (VOCs) in biological samples has emerged as a promising screening approach for various diseases, including CRC [4,5,6,7]. This methodology can potentially provide a non-invasive and cost-effective approach for early detection and diagnosis of CRC. The concept underlying the proposed approach is that VOCs present in human breath are linked to metabolic processes and diseases [8,9,10], and can be found in various biological substances, e.g., urine, feces, tissue, including exhaled breath [11,12,13]. Previous studies suggest that cancer cells release VOCs into the bloodstream, which are then expelled from the body through the alveoli in the lungs and can be detected in exhaled air. Breath analysis has emerged as a promising and non-invasive method for accessing volatilome, despite its complexity and the potential for environmental contamination [14,15]. Numerous studies have identified specific VOCs that are elevated in the breath of patients with various types of cancer, including lung, breast, colorectal, prostate cancer, and others [9,16,17,18,19]. The detection of cancer-specific VOCs in biological fluids is a promising area of research, and several techniques have been employed for their analysis. These include, i.a., gas chromatography-mass spectrometry (GC-MS), ion mobility spectrometry (IMS), proton transfer reaction mass spectrometry (PTR-MS), and electronic nose (e-nose) [20,21].

The analysis of VOCs in biological samples can potentially detect changes that may indicate the presence of CRC at an earlier stage than current screening methods, which could lead to improved outcomes for patients [5]. Several studies in different geographic locations have investigated the potential of VOCs analysis as a CRC screening tool, and the results are promising [4,22,23,24,25,26]. However, the main unresolved issue is the chemical identity of potential markers of CRC. Despite numerous reports embracing different populations, very different markers of CRC are being reported. This is due to the fact that the reliable identification of volatile markers is not trivial because of the complex nature of samples, numerous confounding factors, or interindividual variability. In this context, analyses of volatiles released by cancer tissue is especially attractive, as it offers direct access to the mutated cells. Consequently, it is possible to directly pinpoint cancer-related alterations in their metabolism. Several studies have employed VOCs emitted by tissue samples to identify volatile markers of CRC [27]. For instance, de Vietro et al. [28] investigated VOCs released by CRC and normal tissues and found that the levels of benzaldehyde and indole were higher in the headspace of the cancer tissue compared to the normal one. It must be stressed, however, that the number of patients involved in their study was very small (*n* = 7). Zhu et al. [29] assessed the potential of metabolites in tissue samples obtained from 48 patients to identify CRC. In their study, a set of 17 compounds had significantly different concentrations (*p* < 0.05) between the cancer and paracarcinoma tissues. Eight biomarkers from this set (L-lactic acid, 2,3-butanediol, cholesterol, allose, malic acid, muco-inositol, oleic acid, stearic acid) could differentiate well between cancer tissue and paracarcinoma tissue. However, the identified compounds were semi-, or nonvolatile, thus difficult to detect in gaseous samples such as breath, or urine headspace.

The main goal of this study was to investigate and compare the volatile signatures formed by VOCs released from the CRC and non-cancerous tissues obtained during surgery and, thereby, capture alterations in the tissue metabolism induced by this type of cancer and identify VOCs affected by this type of cancer. For this purpose, headspace solid-phase microextraction (HS-SPME) GC-MS were applied.

## 2. Results and Discussion

### 2.1. Validation Parameters

The validation parameters are shown in Table 1. To determine the limits of detection (LOD), the calculation involved analyzing five consecutive blank signals and applying the method proposed by Huber [30]. The limit of quantification (LOQ) was set as three times the calculated value of the LOD. The LOD ranges from 0.03 to 3 ppb. To assess the precision of the measurements, relative standard deviations (RSDs) were calculated based on a series of five measurements obtained from tissue samples from the same patient. The calculated RSDs ranged from 5% to 19%. These values were deemed satisfactory for the objectives of this study. Additionally, the instrument response was observed to exhibit linearity within the investigated concentration ranges, with coefficients of determination ranging from 0.989 to 0.999.

### 2.2. Volatilomic Signatures of Colorectal Cancer and Non-Cancerous Tissues

**Figure 1:**
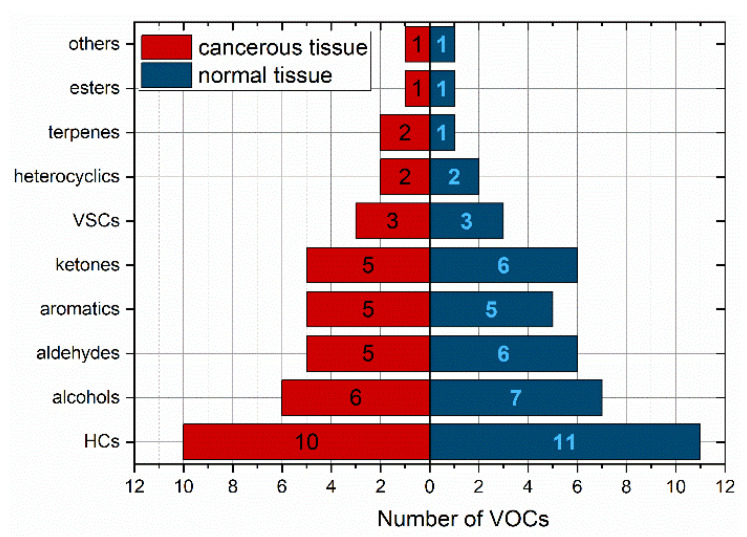
Relative distribution of VOCs with occurrence above 50% according to the chemical classes. HCs—hydrocarbons, VSCs—volatile sulphur compounds.

In total, 163 compounds were detected in the headspace of the tissue samples. More specifically, 149 VOCs were identified in CRC tissues, while 147 VOCs were found in non-cancerous tissues. A total of 138 volatile compounds were present in both cancerous and non-cancerous tissues, but the majority of them were found in very low amounts. Out of these, only 40 compounds (27%) were present in more than 50% of the cancerous tissues. In the case of healthy tissue, this number was 44 (30%). Ten volatiles (2-butanone; dodecane; benzaldehyde; pyridine; octane; 2-pentanone; toluene; p-xylene; n-pentane; 2-methyl-2-propanol) occurred in at least 90% of both types of samples; 1-propanol exceeded this number only in cancer tissue (86% in normal one) and acetone in normal tissue (82% in cancer one). Thereby, these VOCs can be considered as omnipresent. The chemical classes of VOCs with an incidence higher than 50% were depicted in Figure 1 and shown in Table 2.

The distribution of the VOCs according to their chemical classes in both types of samples was similar. The most abundant classes of VOCs in both tissue types were hydrocarbons (25%) and alcohols (15%), followed by aldehydes, ketones, and aromatics. The number of detected compounds per sample varied between subjects and ranged from 28 to 77 (median 49).

### 2.3. Differences between the Volatilomic Signatures of Healthy and Cancerous Tissues

To compare the levels of VOCs between cancerous and non-cancerous tissues, a Wilcoxon signed rank test was employed. The statistical significance threshold was set at *p* < 0.05. However, for certain VOCs where direct quantification was not possible, we assumed a linear detector response for all observed concentration levels. In such cases, their levels were compared solely based on their peak areas. Only VOCs with an incidence above 50% were included in the statistical analysis. Table 3 lists the compounds that showed a significant difference in emission between normal and cancerous tissues.

Among the volatile organic compounds (VOCs) analyzed, fifteen of them displayed consistent differences in their headspace concentrations above the samples under investigation. Out of these, four compounds exhibited increased emissions from cancerous tissue, while the remaining eleven showed reduced release from this type of tissue. The species emitted by cancerous tissue comprised 1-propanol, pyridine, isoprene, and methyl thiolacetate.

Although the metabolic pathways that lead to the production of many VOCs in the human organism are the matter of discussion, there are several pathways that may be responsible for their release by the tissues under study.

Some of the volatiles detected can originate from exogenous sources such as microbiota metabolism, food, environmental exposure, or hospital environment (e.g., propofol). This group also embraces aromatic compounds that are most probably environmental, or smoking-related contaminants.

Alcohols can stem from the following: (i) reduction in aldehydes, or ketones performed by alcohol dehydrogenases (ADHs) [31]; (ii) hydroxylation of branched alkanes [32], and (iii) biotransformation of ethers [33]. The first pathway leads to the production of primary or secondary alcohols. For instance, 3-methyl-1-butanol can be a product of 3-methylbutanal conversion and 2-propanol can be a product of acetone reduction. The hydroxylation of branched alkanes is mediated by cytochrome P450 isoforms (1A2, 2B6, and 2E1), and occurs predominantly at secondary or tertiary C-H bonds. In such a way, 2-methylpropane is converted into 2-methyl-2-propanol [32]. Since 2-methylpropane is too volatile to be detected using the applied analytical method, it is not clear if this species was present in the volatilome of samples under study. Another route for the formation of secondary alcohols involves the biotransformation of aliphatic ethers, facilitated by cytochrome P450 enzymes [12]. In this process, 2-ethoxy-2-methylpropane and 2-methoxy-2-methylpropane could potentially contribute to the production of 2-methyl-2-propanol. However, upon analysis of the headspace of tissues of interest, none of the potential substrates associated with this pathway was detected. The release of 2-ethyl-1-hexanol may also be attributed to the metabolism of di(2-ethylhexyl)phthalate (DEHP). DEHP is a plasticizer widely utilized in polyvinyl chloride products. In the human body, it undergoes rapid hydrolysis, mediated by cholesterol esterase (CEase) and/or carboxylesterase Ces1e, resulting in the formation of mono(2-ethylhexyl) phthalate (MEHP) and 2-ethylhexanol [34]. When it comes to aldehydes, several metabolic mechanisms could be responsible for their production. These include the following: (i) reversible oxidation of alcohols mediated by ADHs and/or cytochrome P450 CYP2E1, and (ii) lipid peroxidation [35]. Production of straight-chain C3–C10 aldehydes was shown to be associated with the peroxidation of polyunsaturated fatty acids of membrane phospholipids induced by reactive intermediates produced under conditions of oxidative stress [35]. Numerous aldehydes can be generated as secondary products during the lipid peroxidation including butanal, pentanal, or nonanal [36]. An optional route for the aldehydes production is the oxidation of alcohols. Thus, the production of pentanal might be associated with the oxidation of 1-pentanol, whereas the emission of nonanal with the bioconversion of 1-nonanol performed by ADHs.

The formation of ketones can be attributed to two possible pathways: (i) the oxidation of secondary alcohols carried out by ADHs and/or cytochrome P450 CYP2E1, and (ii) the β-oxidation of fatty acids. In addition to primary alcohols, ADHs have the capability of oxidizing secondary and cyclic alcohols [37]. Consequently, 2-pentanone may stem from 2-pentanol, acetone can derive from 2-propanol, and cyclohexanone might originate from cyclohexanol. In humans, ketones can also be formed via the *β*-oxidation of the fatty acids. For example, 2-pentanone is proposed to be formed via 𝛽-oxidation of hexanoic acid in the peroxisomal pathway [38]. Possibly other ketones observed within this study could be produced in analogous way. Acetone is a major VOC produced in the human organism with high abundances in bodily fluids. Its major source is endogenous decarboxylation of Acetyl–CoA.

The presence of dimethyl sulfide (DMS) can be linked to the metabolism of sulfur-containing amino acids: methionine and cysteine [39]. Thus, DMS might be produced by thiol S-methyltransferase that can methylate methanethiol, that can be formed from (i) methionine by L-methionine γ-lyase, or (ii) hydrogen sulfide by S-methyltransferase. H_2_S can in turn stem from desulphhydration of cysteine performed by cystathionine-β-synthase (CBS), cystathionine γ-lyase (CSE), and 3-mercaptopyruvate sulphurtransferase.

Of the VOCs that were released, hydrocarbons were the most numerous classes. The emission of n-pentane might mirror peroxidation of unsaturated fatty acids induced by oxidative stress. There is evidence that lipid peroxidation of ω3 and ω6 fatty acids leads to the production of ethane and n-pentane [40,41]. More specifically, these hydrocarbons are generated via β-scission of alkoxy radicals formed by the homolytic cleavage of fatty acids hydroperoxides. For example, n-pentane was demonstrated to be produced from linoleic and arachidonic acids [41]. The metabolic routes leading to the generation of the remaining hydrocarbons are unknown; however, they are hypothesized to be produced during the lipid peroxidation processes.

Fifteen volatiles exhibited alterations in their headspace concentrations above the cancer tissue samples. These changes can be triggered by cancer-related modifications of the metabolic routes. The emission of 2-butanone, 2-pentanone, and cyclohexanone from the cancer tissue was found to be reduced. This finding can indicate the upregulation of the ketone’s metabolism, or downregulation of their production. The metabolism of ketones involves the aforementioned ADHs that can reversibly convert these species into secondary alcohols. On the other hand, the simplest ketone—acetone—is converted in mammals into acetol by cytochrome P450 isozyme CYP2E1 [37]. Acetol, in turn, is further degraded into pyruvate, formate, or acetate. The same enzyme was demonstrated to convert other light ketones in analogous way. Thus, 3-hydroxy-2-butanone was shown to be a product of 2-butanone degradation [42]. Then, 3-hydroxy-2-butanone is next reduced into 2,3-butanediol. Perhaps 2-pentanone and cyclohexanone undergo analogous conversion. If so, the reduced emission of these ketones may indicate the downregulation of the CYP2E1 activity in CRC tissue.

Three alcohols were found to be released in lower amounts from the cancer tissue than from the healthy one. The reduced levels of 2-ethyl-1-hexanol and 3-methyl-1-butanol can reflect upregulated expression of alcohol dehydrogenases. Indeed, the ADH activities were demonstrated to be significantly higher in CRC than in healthy tissues [43]. The degradation of 2-methyl-2-propanol is performed probably via another route. It is metabolized by oxidation into 2-methyl-1,2-propanediol and next into 2-hydroxyisobutyrate, which are eliminated from the body via urine [44]. However, the exact enzymes mediating this conversion are unknown.

Of the hydrocarbons, n-tetradecane and n-tridecane exhibited lowered headspace concentrations above the cancer tissues. The metabolism of hydrocarbons in humans involves cytochrome P450 enzymes (e.g., 1A2, 2B6, and 2E1) and occurs in the case of n-alkanes predominantly at secondary C-H bonds, leading to the formation of secondary alcohols [32]. Thus, in accordance with this pathway n-tridecane might be transformed onto 2-tridecanol.

Four species were emitted in higher amounts by cancerous tissue, namely 1-propanol, pyridine, isoprene, and methyl thiolacetate. The presence of these VOCs in higher amounts in CRC tissue may mirror the altered metabolic pathways that occur in cancerous cells.

Isoprene is one of the most abundant VOCs contained in human breath [45]. Previous studies confirmed that isoprene levels are elevated in patients with chronic renal failure undergoing hemodialysis [46]. In turn, decreased isoprene concentrations have been observed in patients with lung and gastric cancer, chronic liver disease, and heart failure [47,48,49,50]. The origin of isoprene in the human organism is still unclear. In higher eukaryotes, isoprene is believed to be produced from isopentenyl pyrophosphate (IPP) and its isomer dimethylallyl pyrophosphate (DMAPP) in the mevalonic acid pathway [45]. However, there is convincing evidence that muscle tissue acts as an isoprene major production site [51].

Propanol is a kind of alcohol, which is commonly used as a solvent in the production of a variety of products, including pharmaceuticals, cosmetics, and personal care products. However, as mentioned above, alcohols can also be produced in the human body during metabolic processes, and increased levels of alcohols in the breath have been reported in liver disease, including ethanol, methanol, and propanol [52]. It should be noted that there is limited research on the presence of 1-propanol in cancer tissue. The study which focused on gastric cancer examined VOCs released by both cancerous and healthy tissues. Buszewski et al. [53] discovered that gastric cancer tissue had higher concentrations of 1-propanol and CS2 than that of the normal tissue. Similar results were obtained within this study—we found a high concentration of 1-propanol in CRC tissue. Pyridine was also overproduced by CRC tissue. Pyridine is a nitrogen-containing heterocyclic compound that is commonly found in tobacco smoke [54]. Nonetheless, the pyridine ring serves as a building block for several essential compounds in the human body, such as nicotinic acid and nicotinamide. These compounds are forms of vitamin B3 (niacin) and act as precursors to crucial molecules like nicotinamide adenine dinucleotide (NAD+), nicotinamide adenine dinucleotide phosphate (NADP+), as well as their respective reduced forms (NAD(P)H). Previously, our study group discovered that pyridine overproduction was also found in gastric cancer tissue [13,55]. This suggests that pyridine could potentially serve as a biomarker not only for gastric cancer, but also for CRC detection and monitoring. However, the exact role of pyridine in the development and progression of CRC is not well understood and requires further studies.

The metabolic processes underlying the elevated production of methyl thiolacetate are unknown.

Our determined VOCs presence in CRC tissue could be attributed to the altered metabolic pathways that occur in cancerous cells. The identification of specific VOCs emitted in higher amounts by CRC tissue, such as 1-propanol, pyridine, isoprene, and methyl thiolacetate, is a promising area for non-invasive cancer diagnosis and monitoring. However, more research is needed to fully understand the mechanisms behind the production and emission of these VOCs in CRC tissue.

## 3. Materials and Methods

### 3.1. Chemicals and Standards

High-purity liquid substances were used to produce all standard mixtures. Reference chemicals, with purities ranging from 95% to 99.9%, were obtained from Merck (Darmstadt, Germany). To create the standards, a few microliters of liquid compound were injected and evaporated into 1-L glass bulbs (Supelco, Bellefonte, ON, Canada) that were heated and evacuated. Different concentrations of VOCs of interest for calibration were achieved by transferring appropriate volumes from the bulb mixtures into 3–25 L Tedlar bags (SKC Inc., Eighty Four, PA, USA) that contained purified and humidified air with 100% relative humidity at 34 °C. Calibration gas mixtures were created with VOCs volume fractions ranging from 0.07 to 160 parts per billion by volume (ppb). Seven different and independent concentration levels were used to create the calibration curves.

We endeavored to measure the concentrations of volatile organic compounds (VOCs) that showed varying levels in different headspaces. Regrettably, the team faced obstacles in quantifying several compounds. These challenges arose either due to the unavailability of these compounds in their pure form or difficulties in producing reliable reference mixtures, which hindered their ability to conduct thorough analyses.

### 3.2. Study Subjects and Sampling

A cohort of 50 patients aged 35 to 85 (median age 72) diagnosed with CRC were involved in this study. These patients were recruited in the Riga East University Hospital, Latvian Oncology Centre (Riga, Latvia). All the study participants were scheduled for elective colon surgery. Detailed clinical characteristics of the patients are presented in Table 4.

Tissue sampling, sample storage, and transport were based on our group’s previous experience [13,55]. Tissue samples were taken during CRC surgery. The pathologists prepared tissues samples; cancerous tissue and tissue without malignant infiltration were resected from each patient. For the analytical measurements, around 100 mg of both cancerous and non-cancerous tissues were used. Each sample was carefully placed into 2 mL amber glass vials, rapidly frozen in liquid nitrogen, and then stored in a freezer at −80 °C. During transportation to the GC-MS analysis facilities, the samples remained in a frozen state, and dry ice was used to maintain the required temperature conditions. An effort was made to minimize the time of storage (maximal storage time was 6 weeks).

### 3.3. Headspace Solid-Phase Microextraction Sampling Protocol

To extract volatile compounds from tissue samples, headspace solid-phase microextraction (HS-SPME) was used. Before analysis, tissue samples were thawed at 40 °C for 5 min and 100 mg of a frozen sample were placed in a 20 mL silanized glass headspace vial (Gerstel, Germany). The vial was rinsed with high purity nitrogen (99.9999%) and sealed with a septa (butyl/PTFE septa, Gerstel, Germany). HS-SPME was performed automatically (MPS sampler, Gerstel, Germany) using a 75 μm Carboxen-PDMS SPME fiber inserted into the vials and exposed to the headspace gas for 50 min at 37 °C. The fiber was then introduced into the inlet of the GC where the volatiles were thermally desorbed at 290 °C in a splitless mode (0.75 min). Blank samples (pure nitrogen in vials) were analyzed in parallel to detect possible contaminants. If applicable, the concentrations of VOCs in blank samples were subtracted from the respective values in the tissue samples. Samples were analyzed in batches on a daily randomized schedule.

### 3.4. Gas Chromatography–Mass Spectrometry Analysis

The GC-MS analysis was carried out using an Agilent 8890/7079B GC-MS system. The GC injector contained an SPME liner (with an inner diameter of 0.75 mm, supplied by Supelco in Canada) and was maintained at 290 °C and operated in splitless mode for 0.75 min, followed by split mode with a ratio of 1:50. The extracted compounds were separated using an Rxi-624Sil MS column (30 m × 0.32 mm × 1.8 μm, Restek in Bellefonte, PA, USA) under constant helium flow at a rate of 1.4 mL/min. The column temperature program started at 37 °C for 12 min, then ramped up by 5 °C per minute to 150 °C, next ramped up by 10 °C per minute to 290 °C, and finally remained at 290 °C for 8 min. The mass spectrometer operated in the SCAN mode with an associated mass to charge ratio (*m*/*z*) range from 20 to 250. The peak integration process relied on extracted *m*/*z* ratio chromatograms. By using this approach, the majority of the peaks of interest were effectively separated from neighboring compounds. Throughout the analysis, the quadrupole, ion source, and transfer line were carefully maintained at temperatures of 150 °C, 230 °C, and 280 °C.

## 4. Conclusions

The aim of this study was to characterize and compare the volatile signatures formed by VOCs released from the CRC and non-cancerous tissue. The species emitted in higher amounts by cancerous tissue comprised were 1-propanol, pyridine, isoprene, and methyl thiolacetate. The outcomes of this study provide evidence that the VOCs signature of the CRC tissue is modified by the CRC. The volatile constituents of this distinct signature can be emitted through exhalation and serve as potential biomarkers for identifying the presence of CRC. Reliable identification of the VOCs associated with CRC is essential to guide and tune the development of advanced sensor technologies that can effectively and sensitively detect and quantify these markers. The results of this study are a step towards the development of a new non-invasive breath test for the detection of CRC. Further studies are necessary to confirm the credibility of these prospective markers and to acquire a better understanding of their origin and destiny within the human body.

Several limitations of this study should be mentioned. First, the non-cancerous tissue samples could be contaminated with VOCs released by the CRC tissue, since the matched samples originated from the same colon sample taken during the surgery. Consequently, the real differences in the emissions might be reduced, impacting the detection of biomarkers. Secondly, the sensitivity of the GC-MS analytical method may have been influenced by the limited size of the provided tissue samples, which were approximately 100 mg. Finally, sample storage and processing may have impacted the chemical patterns that were obtained. However, the VOCs were found to be stable (within the RSD of the method) in frozen samples over the period of 6 weeks.

## Figures and Tables

**Table 1:** Retention times (R_t_) (min), *m*/*z* of the quantifier ions, LODs (ppb), RSDs (%), coefficients of variation (R^2^), and linear ranges (ppb) for compounds of interest.

VOC	CAS	R_t_ [min]	Quantifier Ion *m*/*z*	LOD [ppb]	RSD [%]	R^2^	Linear Range [ppb]
2-methyl-2-propanol	75-65-0	4.78	59	0.1	5	0.996	0.3–30
1-propanol	71-23-8	6.33	31	3	19	0.996	9–40
ethyl acetate	141-78-6	6.68	43	0.08	6	0.998	0.24–35
2-butanone	78-93-3	7.6	72	0.4	10	0.999	1.3–40
2-pentanone	107-87-9	13.24	86	0.03	10	0.996	0.09–24
pyridine	110-86-1	16.94	52	0.37	6	0.994	1.1–50
3-methyl-1-butanol	123-51-3	17.95	70	0.11	6	0.989	0.33–29
cyclohexanone	108-93-0	25.29	57	0.03	7	0.992	0.09–26
DL-limonene	5989-27-5	29.42	68	0.47	9	0.989	1.5–25
2-ethyl-1-hexanol	104-76-7	30.6	57	0.8	15	0.989	2.8–40

VOC—volatile organic compound; CAS—Chemical Abstracts Service; LOD—limits of detection; RSD—relative standard deviations.

**Table 2:** VOCs with occurrence above 50% according to the chemical classes.

Class	Name (CAS; Occurrence T/N [%])
Hydrocarbons	n-dodecane (112-40-3; 96/100), n-octane (111-65-9; 94/96), n-pentane (109-66-0; 90/98), isoprene (78-79-5; 72/76), n-decane (124-18-5; 68/72), n-undecane (1120-21-4; 62/74), n-nonane (111-84-2; 60/60), n-tetradecane (629-59-4; 58/64), n-hexane (110-54-3; 58/64), dicyclopentadiene (77-73-6; 52/44), n-tridecane (629-50-5; 42/54), 2-methyl-butane (78-78-4; 42/52)
Alcohols	1-propanol (71-23-8; 96/86), 2-methyl-2-propanol (75-65-0; 90/90), ethanol (64-17-5; 88/86), propofol (2078-54-8; 78/76), 2-propanol (67-63-0; 70/70), 3-methyl-1-butanol (123-51-3; 62/84), 2-ethyl-1-hexanol (104-76-7; 40/58)
Aldehydes	nonanal (124-19-6; 78/86), butanal (123-72-8; 62/84), pentanal (110-62-3; 58/52), decanal (112-31-2; 54/62), dodecanal (112-54-9; 46/52)
Aromatics	benzaldehyde (100-52-7; 96/100), toluene (108-88-3; 92/100), p-xylene (106-42-3; 92/100), o-xylene (95-47-6; 80/76), benzonitrile (100-47-0; 74/74), ethylbenzene (100-41-4; 74/72)
Ketones	2-butanone (78-93-3; 96/100), 2-pentanone (107-87-9; 92/100), acetone (67-64-1; 82/96), 2,3-butanedione (431-03-8; 80/86), 6-methyl-5-hepten-2-one (110-93-0; 50/50), cyclohexanone (108-94-1; 34/58)
Volatile sulfur compounds	dimethyl sulfide (75-18-3; 88/86), carbon disulfide (75-15-0, 88/76), methyl thiolacetate (1534-08-3; 58/50)
Heterocyclics	pyridine (110-86-1; 94/98), pyrrole (109-97-7; 70/86)
Terpenes	d-limonene (5989-27-5; 60/74), alpha-pinene (80-56-8; 50/40)
Esters	ethyl acetate (141-78-6; 84/88)
Other	acetoin (513-86-0; 66/74)

CAS—Chemical Abstracts Service, T/N tumor tissue vs. normal tissue.

**Table 3:** Compounds exhibiting differences in emission between normal and cancer tissues. Compounds in italics were not quantified for the reasons described in the text.

VOC	CAS	Change T vs. N	Incidence [%]		Mean [ppb]		*p*-Value
			Tumor	Normal	Tumor	Normal	
2-butanone	78-93-3	↓	96	100	4.1	4.9	0.01
1-propanol	71-23-8	↑	96	86	53	17	0.01
pyridine	110-86-1	↑	94	98	88	44	4.1 × 10^−3^
2-pentanone	107-87-9	↓	92	100	2.4	2.8	0.01
2-methyl-2-propanol	75-65-0	↓	90	90	2.7	6.3	9.8 × 10^−6^
ethyl acetate	141-78-6	↓	84	88	1.1	4.4	8.5 × 10^−5^
*isoprene*	*78-79-5*	↗	*72*	*76*	*nq*	*nq*	*0.03*
3-methyl-1-butanol	123-51-3	↓	62	84	0.39	1.0	4.3 × 10^−3^
d-limonene	5989-27-5	↓	60	74	3	4.5	0.03
*methyl thiolacetate*	*1534-08-3*	↗	*58*	*50*	*nq*	*nq*	*5.9 × 10^−4^*
*tetradecane*	*629-59-4*	↙	*58*	*64*	*nq*	*nq*	*0.04*
*dodecanal*	*112-54-9*	↙	*46*	*52*	*nq*	*nq*	*0.04*
*tridecane*	*629-50-5*	↙	*42*	*54*	*nq*	*nq*	*3.3 × 10^−3^*
2-ethyl-1-hexanol	104-76-7	↓	40	58	5	1.0	0.03
cyclohexanone	108-94-1	↓	34	58	0.12	0.5	0.02

VOC—volatile organic compound; CAS—Chemical Abstracts Service, T—tumor tissue, N—normal tissue, nq—not quantified.

**Table 4:** Clinical features of study subjects.

Gender	*n*	%	Age Range (Median)	CRC Stage				Cancer Differentiation Grade		
				I	II	III	IV	1	2	3
Males	30	60%	35–84 (72)	8	16	5	1	7	20	3
Females	20	40%	50–85 (72)	7	6	6	1	4	12	4
Total	50	100%	35–85 (72)	15	22	11	2	11	32	7

*n*—number of patients, CRC—colorectal cancer.

## Data Availability

The data presented in this study are available upon request from the corresponding authors.

## Sample Availability

Samples of the compounds are not available from the authors.
